# Incorporating patients’ perspectives into the initial stages of core outcome set development: a rapid review of qualitative studies of type 2 diabetes

**DOI:** 10.1136/bmjdrc-2018-000615

**Published:** 2019-02-28

**Authors:** Sarah L Gorst, Bridget Young, Paula R Williamson, John P H Wilding, Nicola L Harman

**Affiliations:** 1 MRC North West Hub for Trials Methodology Research, Department of Biostatistics, University of Liverpool, Liverpool, UK; 2 MRC North West Hub for Trials Methodology Research, Department of Psychological Sciences, University of Liverpool, Liverpool, UK; 3 Obesity and Endocrinology Clinical Research Group, University of Liverpool and Aintree University Hospital, Liverpool, UK

**Keywords:** type 2 diabetes, core outcome set, patient perspectives, qualitative, rapid review

## Abstract

Conducting systematic reviews of qualitative studies to incorporate patient perspectives within the early stages of core outcome set (COS) development can be resource intensive. We aimed to identify an expedited approach to be used as part of the wider COS development process. Specifically, we undertook a rapid review of qualitative studies of patients’ views and experiences of type 2 diabetes. We searched MEDLINE from inception to June 2017 to identify studies reporting qualitative empirical findings of perspectives of people with type 2 diabetes. Qualitative methodological filters were used to minimize irrelevant references. Drawing on content analysis, data synthesis involved identifying text in eligible studies relevant to outcomes of type 2 diabetes and interpreting and categorizing this according to the 38 core domains of the Core Outcome Measures in Effectiveness Trials taxonomy. Of 146 studies screened, 26 were included. Four hundred and fifty-eight outcomes were derived from the included studies. In comparison to the outcomes extracted from clinical trials, more life impact outcomes were derived from the qualitative studies, but fewer physiological/clinical outcomes. Outcomes relating to ‘mortality/survival’ and ‘role functioning’ were more prevalent in studies conducted in low/middle-income countries. This rapid review and synthesis of qualitative studies identified outcomes that had not previously been identified by a systematic review of clinical trials. It also identified differences in the types of outcomes given prominence to in the clinical trials and qualitative literatures. Incorporating qualitative evidence on patient perspectives from the outset of the COS development process can help to ensure outcomes that matter to patients are not overlooked. Our method provides a pragmatic and resource-efficient way to do this. For those developing international COS, our method has potential for incorporating the perspectives of patients from diverse countries in the early stages of COS development.

## Background

Type 2 diabetes mellitus, characterized by abnormal glucose metabolism and an inadequate compensatory insulin secretion response, accounts for over 90% of all cases of diabetes.^1^ Treatment of type 2 diabetes often targets abnormal glucose levels, yet outcomes measured in clinical trials of glucose-lowering interventions are inconsistent^2^ and this heterogeneity in outcomes limits the usefulness of trial findings to patients and other decision-makers.^3 4^ The Selecting Core Outcomes for Randomised Effectiveness trials In Type 2 diabetes (SCORE-IT) study^5^ aims to address these issues by developing a core outcome set (COS) for use in clinical trials of glucose-lowering interventions in people with type 2 diabetes.

COS represent agreed standardized sets of outcomes that should be measured and reported, as a minimum, in all clinical trials for a specific health condition.^6^ The development and implementation of COS can improve the relevance and consistency of trial outcomes and allow the results of clinical research to be pooled and compared, thereby reducing waste in research.^7^ The first step in the development of a COS typically involves a review of existing knowledge (eg, systematic review of outcomes used in previous studies) to inform the consensus process.^8^ In the case of the SCORE-IT study, this has involved a systematic review of outcomes used in registered clinical trials of glucose-lowering interventions for type 2 diabetes.^2^ While this is typical of many COS, clinical trials often overlook the outcomes that are important to patients^9 10^ and so the outcomes identified in such reviews are likely to predominantly reflect the perspectives of researchers and clinicians.^11^ This is a concern, as there is evidence that the input of patients leads to the identification of core outcomes beyond those identified by practitioners alone.^12–15^ Furthermore, the Core Outcome Set-Standards for Development project recently established the inclusion of patient stakeholders within the COS development process to be a minimum standard for COS.^16^

Primary qualitative research is particularly suited to accessing patient perspectives on outcomes, as these studies allow patients to voice their views and experiences in an open-ended way and in their own words.^17^ The findings from such studies can contribute in several ways to COS development including to the ‘long list’ of outcomes needed in the early stages of the process. However, primary qualitative research can be resource intensive and require study team expertise in qualitative methodology.^18^ Systematic reviews of qualitative studies potentially provide an alternative to conducting primary qualitative research where suitable published studies are available.^17^

Previous systematic reviews of qualitative studies have identified outcomes of importance to patients and informed the development of COS in critical illness, bariatric and metabolic surgery, neonatal care and tuberculosis.^19 20^ Such reviews have identified outcomes that were not reported in systematic reviews of clinical trials, indicating that qualitative evidence is needed to ensure that the initial ‘long list’ of outcomes is comprehensive and does not omit outcomes important to patients.^19 21^ However, these previous systematic reviews of qualitative studies have undertaken exhaustive literature searches and are likely to be time consuming and resource intensive.^22^ Systematic reviews inform the early phases of COS and comprise one small aspect of the COS development process. As many developers have limited time and resources, there is a need to identify an expedited approach for identifying outcomes that are important to patients, which can subsequently inform the development of COS. Existing qualitative reviews for a specific condition could be considered; however, in the case of type 2 diabetes, these reviews^23 24^ have not been conducted in the context of COS development and as such group findings into overarching concepts, many of which have little relevance to COS. Here we report an expedited approach for identifying outcomes reported by people with type 2 diabetes when asked about their lived experience.

## Aims

Our aims in undertaking this review were: to identify an expedited approach for incorporating the patient perspective within the initial stages of COS development; to identify outcomes important to people with type 2 diabetes for inclusion in a ‘long list’ of outcomes for the COS consensus process; and to compare outcomes identified from the qualitative literature with those identified via a previous systematic review of outcomes measured in type 2 diabetes clinical trials. We also aimed to compare outcomes identified from qualitative studies of patients living in low/middle-income countries (LMIC) with outcomes identified from qualitative studies conducted in higher income countries (HIC). The prevalence of diabetes in LMICs is high^25^ and it is important to examine if outcomes voiced by patients living in LMICs differ from those of patients in HICs.

## Methods

### Search strategy

Using rapid review methodology, which involves streamlining traditional systematic review methods to synthesize evidence within a shortened time frame,^26^ we searched a single health-related database, MEDLINE, with no date restrictions on 22 June 2017. The search terms, which are indicated in table 1, comprised qualitative methodological filters previously shown to identify qualitative research from the MEDLINE electronic database.^27^ The research field for type 2 diabetes is vast and so search filters designed for maximum specificity^27^ were selected to minimize irrelevant references.

**Table 1:** MEDLINE search strategy

Multifield search		
	(type 2 diabetes OR type II diabetes)	Abstract
AND	patient*	Abstract
AND	(Qualitative OR Themes)	Abstract
AND	(symptom OR treatment OR living with)	Abstract
NOT	(co-morbid* OR foot ulcers OR retinopathy OR nephropathy OR bariatric surgery OR non-alcoholic fatty liver disease OR cardiovascular disease)	Abstract

Studies reporting qualitative empirical findings of the views and experiences of people with type 2 diabetes on their condition and treatment were eligible for inclusion. Type 1 diabetes, gestational diabetes, type 2 diabetes in children and maturity onset diabetes of the young were outside the scope of this review. Studies where the primary focus was the treatment of diabetes comorbidities or complications (eg, diabetic foot ulcer, diabetic retinopathy, nephropathy, bariatric surgery, non-alcoholic fatty liver disease and cardiovascular disease) were also excluded.

### Study selection

SLG and NLH identified and screened titles and abstracts from the MEDLINE search for eligibility, batch checked 10% of these to ensure consistency and discussed uncertainties. There was good agreement between reviewers during the batch check; therefore, the two reviewers each reviewed half the remaining abstracts. Full texts were retrieved and reviewed for articles meeting the following inclusion criteria: participants were people with type 2 diabetes or their partners, the focus was type 2 diabetes and not an associated comorbidity, and qualitative data collection methods (interviews or focus groups) were used. SLG and NLH each reviewed half the full-text papers for eligibility.

### Data extraction

For each included study the following data were extracted:

1. Study aim.
2. Participants (number in study, age, sex, number of years with diabetes).
3. Geographical location of participants.
4. Qualitative data collection methods used.
5. Text excerpts relevant to outcomes.

Our approach to identifying text relevant to outcomes was deductive. It is important to note that none of the qualitative studies explicitly aimed to identify outcomes, although they did contain text that we could interpret as relevant to type 2 diabetes outcomes. Such text comprised any reports about how patients felt or functioned in relation to their diabetes and the healthcare or treatment they had received. Others have previously defined such reports as relevant to outcomes if they describe something that could be used to assess the effect of a healthcare intervention on the patient’s life.^28 29^ We were also guided by this definition. For example, we interpreted the patient quotation, ‘*I just think I like to get my blood glucose inside the right range, as we should’* as about the outcome ‘glycaemic control’. All such text, including participants’ quotations about their views and experiences and the authors’ commentary, was extracted verbatim from both the results and discussion sections of included papers. This text was entered as a separate row in a Microsoft excel spreadsheet (available on request).

SLG and NLH both reviewed and interpreted outcomes from five included studies and checked these for agreement. They each independently reviewed half of the remaining studies.

### Quality appraisal

The role of quality appraisal of qualitative studies in systematic reviews, and whether quality assessment should be used to exclude studies, is debated.^30^ One reviewer (SLG) quality appraised included studies using Critical Appraisal Skills Programme checklist^31^ to facilitate our understanding of them rather than to exclude any studies.

### Data categorization

For the current review, we drew on content analysis to synthesize data from eligible studies. This approach permitted tabulation and frequency counts^32^ and thereby facilitated comparison with the outcomes reported in the previous systematic review of type 2 diabetes clinical trials. We used the Core Outcome Measures in Effectiveness Trials (COMET) taxonomy to categorize text from the studies.

Two reviewers (SLG and NLH) discussed each text excerpt relevant to outcomes to agree how to categorize them, referring back to the original article when necessary to resolve ambiguities. The COMET taxonomy is an outcome classification system suitable for classifying outcomes across all trials, COS, systematic reviews and trial registries^33^ regardless of the condition being investigated. It has been designed to provide high-level differentiation between outcome domains to facilitate uniformity of outcome classification in electronic databases.^33^ Additionally, COS developers have used the taxonomy to assist the classification of outcomes prior to the consensus stage.^34–36^ The taxonomy comprises 38 core domains structured within five top level core areas: death, physiological/clinical, life impact, resource use, and adverse events.

Reviewers categorized text excerpts, considering all 38 core domains of the taxonomy as they did so. Agreement between reviewers (SLG and NLH) was assessed with three batch checks of 10% of all outcomes until 100% agreement was reached. Where uncertainty about an outcome could not be resolved reviewers sought clinical input (JPHW). Where one outcome included multiple components, for example, ‘fear of death’, which encompasses two discrete outcomes ‘fear’ and ‘death’, the outcome was classified under two domains (eg, ‘emotional functioning/well-being’ and ‘mortality/survival’) as recommended.^33^ Outcome categorization was verified by the developer of the COMET taxonomy (SD), who was provided with a list of the outcomes extracted from the included studies, which she categorized blind, without seeing the categorization by the two reviewers. Her outcome categorization was compared with that of the two reviewers and any discrepancies were discussed and resolved.

## Results

### Study characteristics

**Figure 1:**
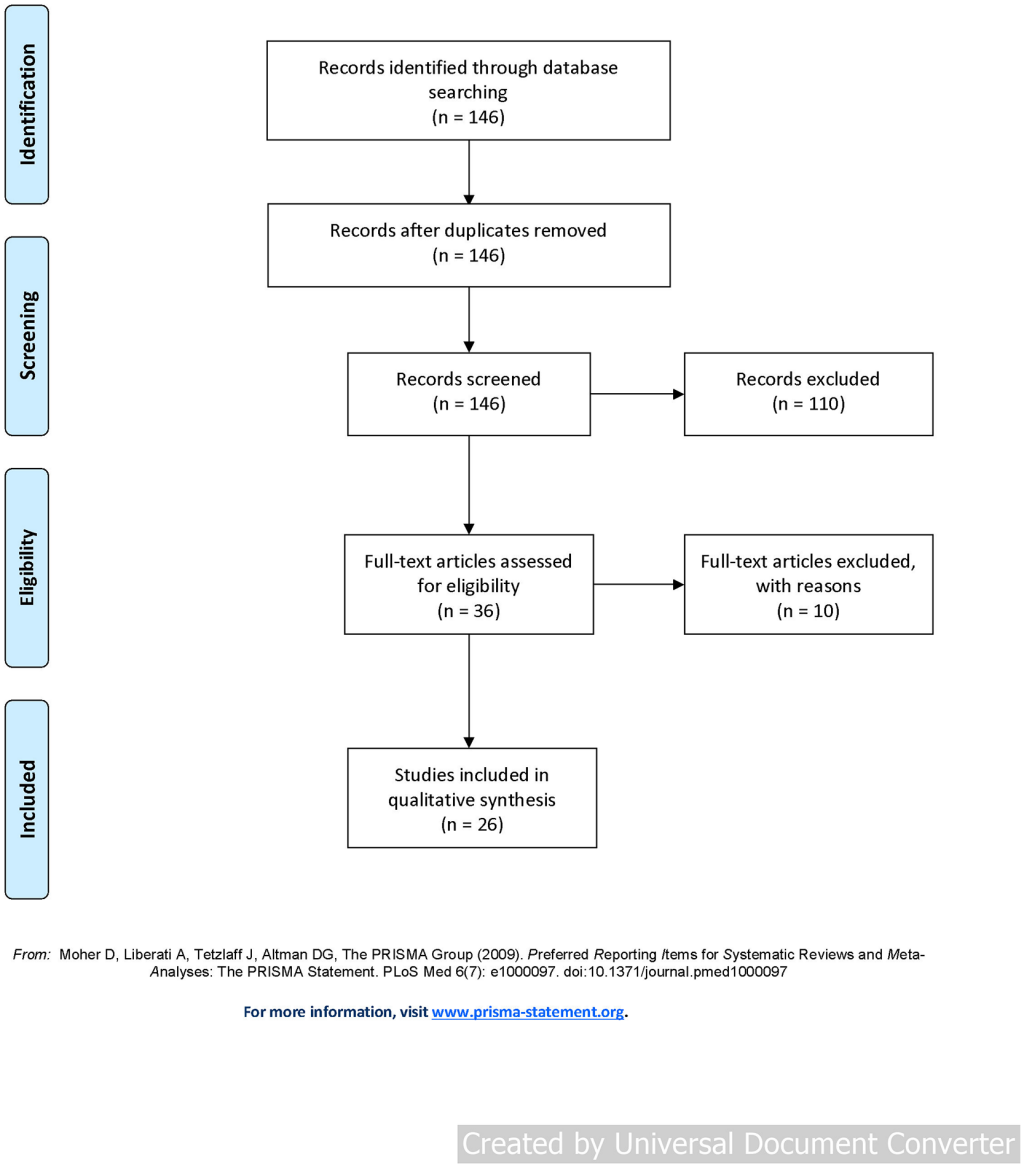
Preferred Reporting Items for Systematic Reviews and Meta-Analyses (PRISMA) flow diagram.

The search returned 146 articles. Of these, 36 were retained after screening titles and abstracts. Following full-text review a further 10 studies were excluded as they did not meet the inclusion criteria. The flow of studies is shown in figure 1.

These 26 included studies involved either qualitative interviews (69%) or focus groups (31%) with a total of 976 patients (median 23 participants, range 5–246) from five continents. All studies included participants with type 2 diabetes with some studies focusing on particular minority groups.^37–42^ Time since diagnosis of diabetes ranged from less than 1 year up to 51 years, although this was not reported in all studies. For one study that also included partners of patients,^38^ content relating to both patients and their partners was included in the synthesis. Another study included both patients and healthcare professionals; however, only data originating from patients were synthesized.^43^ A summary of included studies is provided in table 2.

**Table 2:** Characteristics of included studies

Study ID	Aim	Year of publication	Location of study	Participants (n)	Participant age	Participant sex	Time with diabetes	Data collection method	Outcomes identified (n)
42	Explore behavioral factors affecting what patients do for self-care and why they do it.	1998	USA	51	29–69 years (mean=52.9)	Male: 26 (51%), female: 25 (49%)	0.5–1 year: 4 (8%), 1–5 years: 20 (39%), >6 years: 27 (53%)	Interviews	24
44	Investigate the distress associated with type 2 diabetes.	2002	UK	51	Not reported	Male: 19 (37%), female: 32 (63%)	Not reported	Focus groups	16
60	Explore the views and health beliefs of patients who had experienced a new structured diabetes shared care service.	2003	Ireland	25	30–50 years: 6 (24%); 51–70 years: 13 (52%); >70 years: 6 (24%)	Male: 15 (60%), female: 10 (40%)	Not reported	Focus groups	10
58	Describe personal understandings of illness among patients.	2004	Sweden	44	47–80 years (mean=64)	Male: 23 (52%), female: 21 (48%)	Not reported	Interviews	5
39	Explore the relevance of a reframed model of healthcare consultation.	2004	UK	21	Not reported	Not reported	Not reported	Interviews	21
45	Investigate patients’ perceptions about their illness and treatment strategies to facilitate patient-centered, culture-sensitive clinical skills.	2005	Taiwan	22	44–80 years (mean=60.2)	Male: 12 (55%), female: 10 (45%)	2–25 years (mean=8.3)	Interviews	12
46	Explore the self-reported healthcare goals, factors inﬂuencing these goals, and self-care practices of older patients.	2005	USA	28	65–88 years (mean=74)	Male: 12 (43%), female: 16 (57%)	0–5 years: 11 (39%), >5 to 10 years: 6 (21%), >10 years: 11 (39%)	Interviews	15
47	Explore medication experiences of patients.	2006	USA	138	>70 years: 30 (22%), >50 years: 102 (74%)	Male: 44 (32%), female: 94 (68%)	Mean=13 years	Focus groups	31
48	Identify the obstacles to adherence for patients.	2007	Europe	246	40–80 years (mean=63.8)	Male: 122 (49.6%), female: 124 (49.4%)	1–22 years (mean=10.2)	Focus groups	16
61	Describe the experience of benefit and risk assessment for patients when making treatment decisions.	2007	Canada	18	Mean=60 years	Male: 8 (44%), female: 10 (55%)	Mean=10.7 years	Interviews	29
49	Explore the lived experience of patients converting to insulin therapy.	2007	UK	8	49–72 years	Male: 4 (50%), female: 4 (50%)	Not reported	Interviews	25
50	Understand and document the perspectives of patients regarding the processes and strategies used to self-manage their chronic condition.	2008	Taiwan	41	42–81 years	Male: 22 (54%), female: 19 (46%)	Mean=9.19 years	Focus groups	27
38	Describe cultural and family challenges to illness management in foreign-born Chinese-American patients and their spouses.	2009	USA	40 (20 patients, 20 partners)	Mean=62 years	Male: 16 (40%), female: 24 (60%)	Mean=8.4 years	Interviews	12
51	Explore how women manage their diabetes.	2009	USA	5	50–85 years (mean=70.4)	Female: 5 (100%)	1–18 years (mean=8)	Interviews	16
62	Explore how living with diabetes in everyday life was experienced following a self-management intervention program based on motivational interviewing.	2011	Denmark	22	30–72 years	Male: 10 (45%), female: 12 (55%)	1–11 years	Focus groups	10
43	Explore physicians’ and patients’ views of patients’ difficulty achieving treatment goals.	2012	USA	34 patients, 19 physicians*	43–70 years (mean=59.8)	Male: 20 (59%), female: 14 (41%)	3–51 years (mean=12)	Interviews	10
41	Assess self-management skills of Chinese-Americans.	2012	USA	24 (7 poorly controlled, 17 well controlled)	Poorly controlled: mean=56 years, well controlled: mean=60.6 years	Poorly controlled—male: 3 (43%), female: 4 (57%); well controlled—male: 13 (76%), female: 2 (12%), NA: 2 (12%)	Poorly controlled: mean=6.4 years; well controlled: mean=6 years	Focus groups	11
52	Describe the experiences and ways of coping of older Singaporean Chinese women.	2013	Singapore	10	60–69 years	Female: 10 (100%)	Not reported	Interviews	26
53	Explore the concept of patient values in the context of making decisions about insulin initiation.	2013	Malaysia	21	28–67 years	Male: 12 (57%), female: 9 (43%)	Not reported	Interviews	19
37	Gain a deeper understanding of the difficulties Vietnamese patients experience when accessing services and managing their diabetes.	2013	Australia	15	60 to >70 years: 15 (100%), >70 years: 11 (73%)	Male: 4 (27%), female: 11 (73%)	>1 year: 6, >5 years: 9	Focus groups	24
54	Better understand barriers to glycemic control from the patient’s perspective.	2013	New Zealand	15	33–90 years (mean=63.3)	Male: 5 (33%), female: 10 (67%)	2–30 years (mean=44.3)	Interviews	10
55	Explore the barriers to diabetes control of middle-aged women.	2013	Syria	12	40–65 years	Female: 12 (100%)	4–23 years	Interviews	21
40	Identify issues in self-management, and opportunities for community pharmacies to offer self-management support to these populations.	2013	Australia	24	54–95 years (mean=73)	Male: 10 (42%), female: 14 (58%)	<5 years: 2, 6–10 years: 8, >10 years: 14	Interviews	14
56	Explore patients’ reactions to the diagnosis and their health-related quality of life.	2014	Malaysia	12	50–62 years	Male: 5 (42%), female: 7 (58%)	2.5–21 years	Interviews	32
57	Explore the illness perceptions of patients attending treatment and better understand how they manage their illness.	2016	Ethiopia	39	>70 years: 30	Male: 20 (51%), female: 19 (49%)	1–25 years	Interviews	31
59	Investigate patients’ perceptions and experiences, self-care and engagement with GP-led integrated diabetes care.	2016	Australia	30	<50 to >65 years (mean=60.2)	Male: 16 (53%), female: 14 (47%)	Mean=12 years	Interviews	6

*Data not included in synthesis.

GP, general practitioner; NA, not applicable.

### Quality appraisal

The majority of included studies justified the research design (85%), explained details about the recruitment strategy (81%), took ethical issues into consideration (89%), provided an in-depth description of the data collection (89%) and analysis processes (69%), provided a clear statement of findings (96%), and discussed the implications of the research (81%). In contrast, only a minority of studies adequately described the relationship between the researcher and participants (39%).

### Data categorization

A total of 458 individual outcomes were interpreted from the included studies (median 16 outcomes per study, range 5–32) and categorized according to the COMET taxonomy. Thirty-nine outcomes related to multiple domains and were classified under two or more domains. Thus, 501 outcomes were categorized within the 38 taxonomy domains. Table 3 lists the number of outcomes included in each of the taxonomy domains and the number of studies that included outcomes belonging to each domain.

**Table 3:** Outcome categorization according to the COMET taxonomy

Core area	Core domains	Studies including one or more outcomes in core domain, n (%)	Outcomes included in core domain, n (%)
Death	Mortality/survival	10 (39)	10 (2)
Physiological/clinical	Blood and lymphatic system outcomes	0	0
	Cardiac outcomes	5 (19)	5 (1)
	Congenital, familial and genetic outcomes	0	0
	Endocrine outcomes	1 (4)	1 (<1)
	Ear and labyrinth outcomes	0	0
	Eye outcomes	8 (31)	9 (2)
	Gastrointestinal outcomes	1 (4)	2 (<1)
	General outcomes*	19 (73)	42 (8)
	Hepatobiliary outcomes	1 (4)	1 (<1)
	Immune system outcomes	0	0
	Infection and infestation outcomes	3 (12)	3 (1)
	Injury and poisoning outcomes	1 (4)	1 (<1)
	Metabolism and nutrition outcomes	26 (100)	63 (13)
	Musculoskeletal and connective tissue outcomes	1 (4)	1 (<1)
	Outcomes relating to neoplasms: benign, malignant and unspecified (including cysts and polyps)	0	0
	Nervous system outcomes	4 (15)	4 (1)
	Pregnancy, puerperium and perinatal outcomes	0	0
	Renal and urinary outcomes	11 (42)	13 (3)
	Reproductive system and breast outcomes	0	0
	Psychiatric outcomes	5 (19)	7 (1)
	Respiratory, thoracic and mediastinal outcomes	1 (4)	1 (<1)
	Skin and subcutaneous tissue outcomes	0	0
	Vascular outcomes	10 (39)	12 (2)
Life impact	Social functioning	14 (54)	28 (6)
	Role functioning	16 (62)	20 (4)
	Physical functioning	24 (92)	72 (14)
	Emotional functioning/well-being	24 (92)	106 (21)
	Cognitive functioning	12 (46)	15 (3)
	Global quality of life	3 (12)	3 (1)
	Perceived health status	5 (19)	5 (1)
	Delivery of care	18 (69)	43 (9)
	Personal circumstance	7 (27)	12 (2)
Resource use	Economic	0	0
	Hospital	0	0
	Need for intervention	9 (35)	10 (2)
	Societal/carer burden	2 (8)	2 (<1)
Adverse events	Adverse events/effects	8 (31)	10 (2)

*The COMET taxonomy defines ‘general outcomes’ to include those affecting the whole body, which cannot be attributed to a certain body system, for example, fatigue, malaise, pain (unspecified, not associated with a particular body system), fever (not attributable to infection), anthropometric measures (eg, weight), ‘global’ measures, ‘symptoms’ (not associated with a particular body system), ‘physical health’ and fitness.^45^

COMET, Core Outcome Measures in Effectiveness Trials.

Of the 501 outcomes, 10 (2%) concerned death, 165 (33%) were physiological/clinical, 304 (61%) were associated with life impact, 12 (2%) related to resource use and 10 (2%) pertained to the adverse events core area. Most outcomes fell within the core domains of ‘emotional functioning/well-being’, ‘physical functioning’ and ‘metabolism and nutrition’. Outcomes relating to each of these three domains were identified in more than 90% of included studies and, when combined, comprised 48% of the total outcomes.

### Emotional functioning/well-being

Over one-fifth of the derived outcomes related to ‘emotional functioning/well-being’ (n=106). These outcomes were identified in 24 of the 26 included studies (92%). In 16 studies, patients described being fearful,^41 42 44–57^ with fears relating to medication side effects, treatment escalation, needing insulin injections, dying, uncertainty about the future, and developing complications, such as foot damage, paralysis and loss of eyesight. Relatedly, 10 studies^39 42 47–50 52 55–57^ described patients feeling worried or anxious about symptoms, complications, health deterioration, and ultimately dying prematurely as a result of their diabetes. Patients also commented on how diabetes was a burden^44 47 58 59^ and reported experiencing aggression and frustration,^38 39 43 47 49 51 56 57 59–61^ sadness and depression,^37–39 43 47 52 56 59^ guilt^43 44 54^ and hopelessness.^43 44 52 54^

### Physical functioning

A total of 72 (14%) outcomes concerning physical functioning were derived from 24 included studies (92%). Exercise was identified as an outcome in 16 studies, with patients acknowledging the importance of regular exercise and the benefits it brings; however, some patients labelled it a burdensome activity, which they had difficulty engaging in.^37 39 40 42–46 48 50 52 53 56–58 61^ In 15 studies, patients referred to the self-monitoring activities they engaged in to manage their diabetes, for example, regularly checking blood glucose levels,^37–41 44 47 49 50 52 55–57 61 62^ with many emphasizing that the need for a strict self-management regime had become a burden on their lives. Dietary restrictions were a common difficulty relating to self-management, with many patients articulating a desire for dietary freedom, where they could eat what they want, when they want.^37 38 44 46 49 57 61^

### Metabolism and nutrition

Outcomes relating to metabolism and nutrition were identified in all of the 26 included studies, with 63 (13%) outcomes identified in total. In 20 studies, patients made references to their diet, explaining how healthy eating was necessary for controlling their blood sugar levels.^37 39–43 46 48 50–56 58–62^ In 17 studies, patients spoke about blood glucose-level fluctuations, the importance of glycemic control and the consequences of blood glucose levels falling outside the appropriate range.^38 39 41–43 45–47 49–51 53 55–57 60 62^ Relatedly, 12 studies referred to hypoglycemia, including patients’ concerns over what would happen if they did experience a hypoglycemic episode, fears about the physical symptoms and the steps they would take to avoid hypoglycemia.^37 39 41 44 47 49 52–54 56 57 61^

### Outcomes identified from studies conducted in LMICs

Of the 26 included studies, four (15%) were conducted in LMICs.^63^ These four LMIC studies included one upper middle-income country (Malaysia),^53 56^ one lower middle-income country (Syria)^55^ and one least developed country (Ethiopia).^57^ The most prevalent outcome domains among the LMIC studies were ‘mortality/survival’, ‘general outcomes’, ‘metabolism and nutrition’, ‘role functioning’, ‘physical functioning’, ‘emotional functioning/well-being’ and ‘delivery of care’. Outcomes relating to these seven domains were derived from 100% of the LMIC studies. Five of these seven domains were also among the most reported in the studies conducted in HICs; however, outcomes associated with the ‘mortality/survival’ and ‘role functioning’ domains were less frequently reported in HIC studies, at 27% and 55% of studies, respectively. Additionally, outcomes relating to two of the domains, ‘endocrine outcomes’ (eg, pancreatic function) and ‘hepatobiliary outcomes’ (eg, liver complications), were only derived from the LMIC studies.

### Comparison with outcomes identified in systematic review of clinical trials

We compared the outcomes derived from the qualitative studies with the outcomes identified from the recent systematic review of type 2 diabetes registered clinical trials.^2^ Table 4 shows the number of outcomes identified from both reviews according to the five core areas of the COMET taxonomy. In total, 1446 outcomes were identified from the clinical trials review and 458 outcomes from the review of qualitative studies. Both reviews identified a similar proportion of outcomes related to death, resource use and adverse events. However, the systematic review of clinical trials identified a higher proportion of physiological/clinical outcomes (84% vs 33%), whereas the qualitative studies identified a greater proportion of life impact (61% vs 10%) outcomes. Several domains were only identified in one or other of the reviews. Outcomes relating to the ‘blood and lymphatic system’, ‘immune system’, ‘skin and subcutaneous tissue’, ‘economic resource use’ and ‘hospital resource use’ were only extracted from the clinical trials, whereas outcomes associated with ‘injury and poisoning’ (eg, injuries associated with insulin injections), ‘personal circumstances’ (eg, patients’ support networks) and ‘societal/carer burden’ (eg, patients wanting to be independent and not wanting to be a burden to their family) were only identified in the qualitative studies.

**Table 4:** Number of outcomes identified in the systematic review of clinical trials and synthesis of qualitative literature according to the five core areas within the COMET taxonomy

	Outcomes identified in systematic review of clinical trials, n (%)	Outcomes identified in synthesis of qualitative literature, n (%)
Death	3 (<1)	10 (2)
Physiological/clinical	1221 (84)	165 (33)
Life impact	145 (10)	304 (61)
Resource use	31 (2)	12 (2)
Adverse events	46 (3)	10 (2)

COMET, Core Outcome Measures in Effectiveness Trials.

## Discussion

This review has identified an expedited approach for incorporating patient perspectives within the early stages of COS development. In contrast to previous similar reviews, which have involved exhaustive searches,^19 20^ the streamlined nature of the current review enabled rapid identification of patient-centered outcomes that can be used to contribute to the development of the ‘long list’ of outcomes to inform the COS consensus process.

To our knowledge, this is the first review of qualitative studies to identify outcomes that are important to people with type 2 diabetes. The findings will be used, alongside a review of outcomes measured in clinical trials,^2^ in the development of a COS for type 2 diabetes. Importantly, this review of qualitative studies has identified outcomes that have not previously been measured in type 2 diabetes clinical trials. Without this review, these outcomes would not have been identified for inclusion in the ‘long list’ of outcomes to go forward to the Delphi study.

Most outcomes identified from the qualitative studies related to life impact, whereas in the review of registered clinical trials a relatively small proportion of outcomes related to life impact.^2^ If clinical trial reviews are used as the only source for developing ‘long list’ of outcomes for COS consensus processes, life impact outcomes may become sidelined in favor of outcomes more frequently measured in clinical trials. This is reflected in the COMET database, where far fewer COS encompass life impact outcomes, in contrast to many COS that encompass physiological/clinical outcomes.^33^ Thus, the current review supports the recommendation by Dodd and colleagues^33^ for COS developers to give greater attention to outcomes of life impact. It also illustrates how conducting reviews of qualitative studies can help, alongside other steps such as including patients as participants in COS studies and involving patients and the public in the design of such studies, to ensure COS reflect outcomes that matter to patients.

Given uncertainties about how far COS are applicable beyond those countries that the participants in the development process have been drawn from, it is striking that 84% of published COS studies have not included any participants from LMICs.^64^ While few qualitative studies had been conducted in LMICs, our review has enabled us to compare outcomes identified in LMIC and HIC studies. Outcomes relating to ‘mortality/survival’ were identified in all LMIC studies, yet almost three-quarters of HIC studies made no references to these outcomes. This is most likely due to the higher prevalence of diabetes-related deaths in LMICs.^25^ Similarly, all LMIC studies reported outcomes relating to ‘role functioning’ (eg, ability to work and managing family responsibilities) while almost half of the HIC studies made no references to these outcomes. Being unable to function in one’s life roles is likely to be more detrimental to patients living in LMICs.^65^ Furthermore, three domains relating to diabetes-related complications were only reported in the LMIC studies. Complications are expensive to treat in LMICs, which represent less than 20% of the world’s diabetes care-related expenditure.^66^ It is possible that national economic factors influence which outcomes are important to patients, indicating the importance of ensuring that the perspectives of patients in LMICs are incorporated into COS development processes.

### Strengths and limitations

This study has identified an expedited approach for incorporating the patient perspective into the initial stages of COS development. Our review has enabled evidence from a large number of patients living in a diversity of countries to contribute to the early stages of COS development. We anticipate that such reviews could be performed by COS developers without specialist expertise in qualitative methods, although relevant training would be helpful. Additionally, the method is less resource intensive than other methods for reviewing qualitative evidence which makes it feasible as part of a wider COS process. However, there are some methodological limitations. In line with our expedited approach and recommendations from previous studies,^67 68^ we only searched one database (MEDLINE) and did not search gray literature. While this approach may mean that some relevant studies may have been missed, our aim was not to provide a comprehensive overview of all research relating to patients’ views and perceptions of type 2 diabetes. Rather, to provide an expedited approach for identifying outcomes that are important to patients, as part of a wider process to incorporate the patient perspective in COS development. In comparison to other qualitative systematic reviews we identified few articles for screening, which reflects the specificity^27^ of our search terms. Despite these limitations, the number of studies included in this rapid review is comparable to other reviews of qualitative evidence.^19 20^

A further limitation is that our inclusion of studies relating to a specific experience (eg, patients’ reactions to their diagnosis)^56^ or intervention (eg, the views of patients who had experienced a new structured diabetes shared care service)^60^ may have impacted on the outcomes identified. However, when extracting data from such studies we focused on patients’ general views and experiences of diabetes; we did not extract data that focused solely on patients’ perspectives of specific experiences or interventions. An additional limitation is that while many studies in our review were inductive, our approach to reviewing them and deriving outcomes was deductive. Specifically, categorizing text excerpts according to the COMET taxonomy may have transformed their meaning or diluted the patient perspective. However, this categorization enabled us to compare the different literatures in a common ‘currency’, which was key to identifying differences in the types of outcomes given prominence in the clinical trials versus the qualitative literatures, and differences in the qualitative studies from LMICs and HICs. Finally, our review was largely aggregative, collecting findings of previous studies and describing these according to a predefined taxonomy to allow comparison and address specific aims, rather than a configurative review which seeks to interpret the experiences of patients or generate new theory about these.^69^ Nevertheless, we hope the pragmatic and resource-efficient nature of our method helps the field of COS development by making it easier to incorporate patients’ perspectives.

## Conclusion

This rapid review of qualitative studies identified outcomes that are important to people with type 2 diabetes and its findings will inform the development of a COS for clinical trials of glucose-lowering interventions in people with type 2 diabetes. These patient-derived outcomes contrasted with those identified from a systematic review of clinical trials, pointing to the importance of incorporating patient perspectives from the outset of COS development. Additionally, this review also emphasized the importance of ensuring that patients in LMICs are able to input into the development of COS.
